# The effect of eligibility for antiretroviral therapy on body mass index and blood pressure in KwaZulu-Natal, South Africa

**DOI:** 10.1038/s41598-021-94057-z

**Published:** 2021-07-19

**Authors:** Aditi Kuber, Anna Reuter, Pascal Geldsetzer, Natsayi Chimbindi, Mosa Moshabela, Frank Tanser, Till Bärnighausen, Sebastian Vollmer

**Affiliations:** 1 Department of Economics, University of Goettingen, Göttingen, Germany; 2 Division of Primary Care and Population Health, Department of Medicine, Stanford University, Stanford, CA USA; 3 Africa Health Research Institute, Durban, South Africa; 4 School of Nursing and Public Health, University of KwaZulu-Natal, Durban, South Africa; 5 Lincoln International Institute for Rural Health, University of Lincoln, Lincoln, UK; 6 Centre for the AIDS Programme of Research in South Africa (CAPRISA), University of KwaZulu-Natal, Durban, South Africa; 7 Heidelberg Institute of Global Health (HIGH), Faculty of Medicine, University of Heidelberg, Heidelberg, Germany

**Keywords:** HIV infections, Risk factors, Public health, Health policy

## Abstract

We use a regression discontinuity design to estimate the causal effect of antiretroviral therapy (ART) eligibility according to national treatment guidelines of South Africa on two risk factors for cardiovascular disease, body mass index (BMI) and blood pressure. We combine survey data collected in 2010 in KwaZulu-Natal, South Africa, with clinical data on ART. We find that early ART eligibility significantly reduces systolic and diastolic blood pressure. We do not find any significant effects on BMI. The effect on blood pressure can be detected up to three years after becoming eligible for ART.

## Introduction

In 2019, 38 million people were estimated to be living with HIV, more than half of them living in Eastern and Southern Africa^1^. In this region, about 70% of the people living with HIV (PLHIV) had access to antiretroviral treatment (ART) in 2019^1^. ART increases life expectancy drastically^2–4^, such that its expansion will trigger a change in the age composition of PLHIV^5^. In 2007, adults aged 50 and above already constituted 14% of PLHIV in sub-Saharan Africa^6^. If the present treatment coverage rates remain constant, the number of PLHIV aged 50 + will triple over the coming years^7^.

The expansion of ART and upsurge in life-expectancy of PLHIV might impact the burden of cardiovascular disease (CVD)^8^: As age is an important risk factor for CVD, the burden will increase with the aging of PLHIV. Additionally, observational studies indicate that PLHIV might be subject to a higher risk of CVD than their counterparts^9–11^. Potential reasons are direct impacts of the virus on the body, unhealthy risk factors, comorbidities, or an association with the geographical variation of CVD risks^11^. ART might mitigate the impact of HIV on CVD risks by viral suppression, but ART drugs might also increase CVD risks directly. Observational studies show an increased CVD risk for patients on ART compared to ART naïve PLHIV, especially for protease inhibitor-based regimen^9^. However, the START randomized controlled trial suggests that the overall impact of early compared to deferred ART on CVD risk factors might be clinically negligible within the first years^12,13^.

This study focuses on two intermediate risk factors of CVD; body mass index (BMI) and blood pressure. Similar to CVD risk, systematic literature reviews show that patients on ART have higher BMI and blood pressure measurements, and higher odds of obesity and hypertension compared to ART naïve patients^14–16^. The risk of developing hypertension seems to increase with duration of ART^17,18^. Contrastingly, the START trial did not detect any significant impact of early compared to deferred ART on blood pressure or hypertension, but a small reduction in the use of blood pressure lowering drugs^13^. Also, systematic literature reviews on sub-Saharan Africa do not find a significant association between ART and blood pressure or hypertension^19,20^.

We add to this literature by investigating a natural experimental setting, in which we can estimate the causal effect of early ART eligibility on individual’s BMI, systolic blood pressure (SBP) and diastolic blood pressure (DBP). To this end, we employ a quasi-experimental technique which combines characteristics of observational studies and randomized controlled trials^21^: Observational data from a large population-based sample allows us to analyze the ‘real-life’ effect of ART eligibility. While causal inference in an observational study usually requires strong assumptions such as unconfoundedness, our quasi-experimental design allows us to replace this with weaker assumptions, such that we can plausibly identify the causal effect of early ART eligibility^22^. Thus, our observational data gives us the advantage of a real-life context, while the quasi-experimental nature brings in an internal validity comparable to RCTs^21^.

We exploit the fact that ART eligibility was determined by national guidelines during our observation period. Individuals with a CD4 cell count $\le$ 200 cells/μl were eligible for ART, while individuals with a higher CD4 count were asked to return for a reassessment after 6–12 months (with the exception of patients with TB, WHO stage IV, or pregnant women). In general, eligible patients might not be comparable to ineligible patients: For instance, the time from HIV infection to linkage-to-care is longer for men^23^, and they initiate ART at lower CD4 counts than women^24^. However, patients presenting with similar CD4 counts can be assumed to be on average comparable to each other. Hence, individuals presenting with a CD4 cell count slightly below 200 cells/μl are plausibly similar to individuals with a CD4 count slightly above 200 cells/μl, except for the fact that the former received the possibility to initiate ART, while the later had to return after some time. Under certain assumptions, comparing the outcomes of these two groups gives us the causal effect of early versus deferred ART eligibility.

## Methods

### Data sources and procedures

We use data from a Demographic Information System run by the Africa Health Research Institute. The surveillance area is located in the Hlabisa sub-district of uMkhanyakude in KwaZulu-Natal^25^. The first round of data collection was conducted in 2000. Since 2003, data is collected every 6 months^25^. Individuals are included in the survey if they are considered as a member by one or more households within the surveillance area and have spent at least one night in this household within the past 12 months^25^.

During the surveillance period in 2010, a cross-sectional anthropometric survey was conducted in a random subsample of 30 areas^26^. All residents of the surveillance area > 15 years of age were eligible^27^. WHO STEPS protocol was followed to measure height, weight and blood pressure. The survey addressed 4,608 individuals, of which 55% agreed to blood pressure and 45% to height and weight measurement^26^.

We received data on ART eligibility from a linked clinic-based database. The database tracks laboratory data and clinic visits of HIV-positive individuals who accessed services at one of the 17 public health clinics within the surveillance area since 2007^28^. Data on ART and CD4 counts prior to 2007 was collected retrospectively. CD4 counts were measured at each clinic visit^28^.

From 2004 to August 2011, adults with a CD4 count of $\le$ 200 cells/μl or WHO stage IV HIV (pregnant women: WHO stage III) were eligible for ART, following national and WHO guidelines^29^. Pregnant women and patients with tuberculosis were eligible with a CD4 count of $\le$ 350 cells/μl since April 2010^29^. Ineligible patients were asked to return to the clinic within six months if their CD4 count was $\le$ 500 cells/μl, else within one year^28^. Less than half of the patients which were ineligible returned within a year after the first clinic visit^30^. Subsequent guidelines increased and finally abolished the threshold, but are not covered by our data.

Only individuals 15 years or older and with a non-missing value for at least one of the pairs height/weight, second/third SBP, or second/third DBP were considered. Patients who had been on ART treatment previously were excluded. As there was a substantial loss in care of patients not yet eligible^30^, we only consider an individual’s earliest CD4 cell count. Also, only clinical data recorded between January 2007 (the first date of the database tracking) and the date of the anthropometric data collection were considered. Duplicates were dropped. The final sample consisted of 1132 patients. Data-driven optimal bandwidths as described in the statistical analysis reduced this number to 241 for the BMI analyses and 165 for the blood pressure analyses.

### Ethical approval

The data is secondary to the authors. Primary data collection was approved by the University of KwaZulu-Natal's Ethics Committee and administered by the Africa Health Research Institute. Informed consent was obtained from all study participants. All methods were carried out in accordance with relevant guidelines and regulations. Access to the anonymized data was granted by the Africa Health Research Institute for the purpose of this study.

### Outcomes and covariates

We analyze the data for three outcomes, BMI, SBP, and DBP. We use data on weight and height collected in the anthropometric survey to calculate BMI scores by dividing weight (in kilogram) by squared height (in meters). Due to missing observations for either of the two variables, BMI scores could be obtained for 943 individuals (83%) only. For SBP and DBP, the average of the second and third measurement is calculated, as advised by the STEPS protocol. The average SBP and DBP could be calculated for 1,106 individuals (98%).

Eligibility is defined as having a first CD4 cell count of $\le$ 200 cells/μl, following the national treatment guidelines at that time^29^. Deviation from this eligibility threshold was calculated as the difference between the first CD4 cell count and the threshold. Initiation of ART was defined as uptake of ART within 6 months after the first CD4 count test and before the survey date.

We use information on education years, age, squared age, sex, and years since the first CD4 test for balance checks and as controls in some of the regressions. Data on education was captured during another survey round in the same year.

### Statistical analysis

We employ a regression discontinuity design to examine the effect of ART eligibility on BMI and blood pressure. At the time of the anthropometric survey, national guidelines created a threshold which divided patients based on their CD4 count into ART eligible and ineligible individuals. Under certain assumptions, this threshold is arbitrary to patients with a CD4 count very close to the threshold. For example, patients presenting with a CD4 count of 201 cells/μl and patients presenting with a CD4 count of 199 cells/μl are assumed to be on average very similar with respect to their clinical condition and sociodemographic background. It is assumed that the only difference between the two groups is that to the latter, ART was offered, while the former was advised to return after six months. Thus, any difference we see in BMI and blood pressure across the two groups is plausibly attributable to the offer of ART^31^. The random noise in CD4 count measurement further supports this assumption of a quasi-random assignment of eligibility in a small bandwidth around the threshold^31^. However, in the absence of ART, the CD4 counts of PLHIV will decrease over time, bringing formerly non-eligible patients to the lower side of the threshold. Thus, any estimated difference will reflect the effect of early versus deferred ART eligibility.

The identification strategy hinges on the assumption that the groups just below and just above the threshold do not differ systematically except for their eligibility status. We employ two falsification tests by running a density check around the threshold following the procedure by Cattaneo et al.^32^ and a balance check using t-tests.

Similarly, the choice of the bandwidth around the threshold is crucial. Within a very small bandwidth, e.g., comparing individuals with a CD4 count + /− 1 CD4 cells/μl around the threshold, the individuals are convincingly similar, and the assumptions more likely to hold. At the same time, very small bandwidths contain only few observations, such that the power to detect any meaningful effects might be too small. To identify the optimal bandwidth around the threshold, we use a data-driven procedure minimizing the mean squared error (provided by the user-written Stata package rdrobust)^33^. For BMI, the optimal bandwidth is estimated at + /− 64 CD4 cells/μl around the threshold, reducing the estimation sample to 241 individuals. For SBP and DBP, the optimal bandwidth is estimated at + /− 40 CD4 cells/μl, reducing the estimation sample to 165 individuals.

As eligibility did not predict treatment uptake perfectly, we apply a reduced-form estimate to obtain the intention-to-treat effect at the threshold. We employ a linear functional form of the deviation of the CD4 count from the threshold to allow for a linear trend in our outcomes as the CD4 count increases. More specifically, our main estimation reads as follows:

1 $${y}_{i}={\beta }_{0}+{\beta }_{1}{E}_{i}+{\beta }_{2}{D}_{i}+{\beta }_{3}{E}_{i}\cdot {D}_{i}+{\epsilon }_{i}$$

Here, ${y}_{i}$ denotes the individual’s BMI, SBP or DBP at the time of the visit in 2010, ${E}_{i}$ denotes eligibility (i.e., whether the CD4 count was below the threshold) and ${D}_{i}$ is the deviation from the threshold. ${\beta }_{1}$ estimates the effect of early versus deferred ART eligibility.

In addition to the main specification, we analyze the robustness of the results to the inclusion of control variables using education, age, age squared, sex, and years since the CD4 test. We test for robustness to different bandwidths and to more flexible forms by employing higher-order polynomials. Finally, we analyze heterogeneous treatment effects by sex and over time using the following specification:

2 $${y}_{i}={\beta }_{0}+{\beta }_{1}{E}_{i}+{\beta }_{2}{D}_{i}+{\beta }_{3}{E}_{i}\cdot {D}_{i}+{\sum }_{k}{\beta }_{k1}{E}_{i}\cdot {X}_{i}^{k}+{\sum }_{k}{\beta }_{k2}{X}_{i}^{k}+{\epsilon }_{i}$$

Here, ${X}_{i}^{k}$ denotes binary variables for each year since the CD4 test, respectively for sex. Thus, ${\beta }_{k1}$ estimate the heterogeneous treatment effect by year since the CD4 test, respectively by sex. To obtain the total treatment effect for each distinct group, we test the linear combination of ${\beta }_{1}$ and ${\beta }_{k1}$ for joint significance using the lincom command. All other variables are defined as above. For all regression specifications, heteroskedasticity-robust standard errors are computed. All analyses are conducted with Stata 15.1 SE.

## Results

### Descriptive statistics

Balance checks for a bandwidth of + /− 50 CD4 cells/μl are depicted in Table 1. By design, ineligible and eligible participants differ according to their CD4 count. The two groups are statistically indistinguishable regarding all other covariates except for the years since the earliest CD4 test. On average, the CD4 test of eligible patients occurred three months earlier than the test of non-eligible patients. BMI, SBP and DBP do not differ between both groups in the simple means comparison.

**Table 1:** Balance checks for the ± 50 CD4 cells/μl bandwidth.

	Not eligible			Eligible			
	N	Mean	SD	N	Mean	SD	p-value
Female	98	0.83	0.38	123	0.85	0.36	0.7055
Age	98	38.14	11.88	123	36.92	10.72	0.4223
Education years	92	7.54	4.27	116	8.07	3.98	0.3611
Years since earliest CD4 test	98	2.40	1.01	123	2.66	0.95	0.0502
CD4 count at earliest CD4 test	98	228.95	12.97	123	174.92	14.68	0.0000
BMI	83	25.38	5.65	104	24.46	5.32	0.2525
SBP	98	117.62	18.54	118	117.13	15.88	0.8341
DBP	98	80.62	12.76	118	79.17	11.69	0.3844

**Figure 1:**
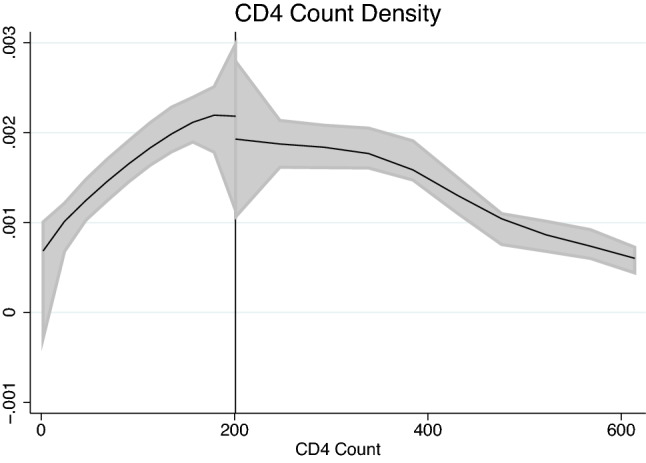
Estimated density of first CD4 counts with 95% confidence interval. The threshold lies at 200 CD4 cells/μl. The null hypothesis of a joint distribution could not be rejected (t = − 0.2028, p = 0.8393).

Figure 1 depicts the estimated density of CD4 counts around the threshold. Using the test developed by Cattaneo et al. ^32^, we cannot reject the null hypothesis of a joint distribution.

**Figure 2:**
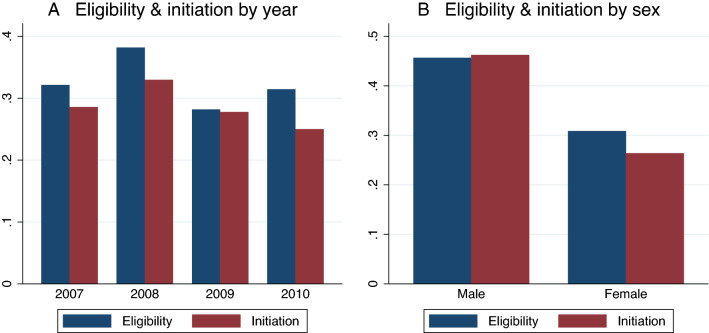
Share of individuals becoming eligible and starting treatment by year and sex.

The distribution of eligibility and treatment uptake over years and by sex are depicted in Fig. 2. The share of patients with a first CD4 cell count $\le$ 200 cells/μl was especially high in 2008. Only a fraction of the patients tested in 2010 already started ART by the time of the household visit in the same year. On average, men were more likely than women to become eligible at the first test.

**Figure 3:**
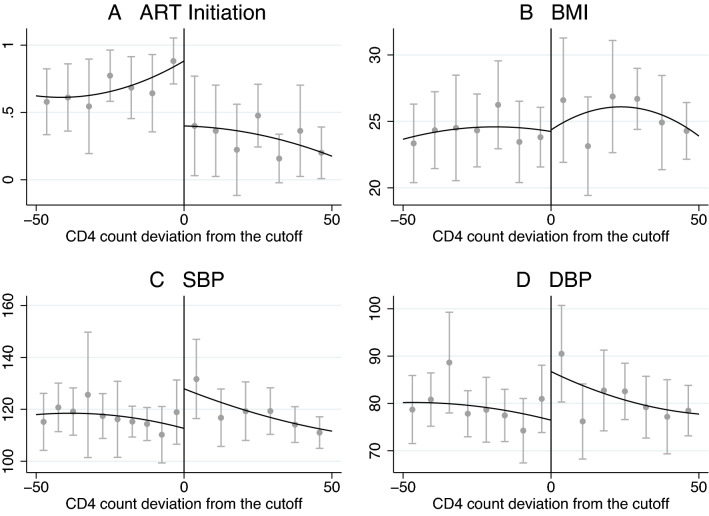
Mean treatment initiation, BMI, SBP and DBP by CD4 cell count bins with 95% confidence intervals. The approximation is a second-order polynomial function differing on both sides of the threshold.

Figure 3 depicts the distribution of treatment initiation, BMI, SBP and DBP over CD4 counts around the threshold. There is a clear jump in treatment probability at the threshold. Moreover, the polynomial fits suggest a reduction in SBP and DBP at the threshold.

### Main results

Table 2 depicts the main estimates from Eq. (1) and the estimates using controls. We find no significant effect of ART eligibility on BMI in any of the specifications. However, there is a significantly negative effect of early ART eligibility on SBP and DBP (p < 0.05). On average, eligible patients have a 15.5 mmHg lower SBP and a 11.1 mmHg lower DBP compared to ineligible patients in the bandwidth around the threshold. This result is robust to the inclusion of socio-demographic controls.

**Table 2:** Estimates for linear specifications with and without controls. The controls are years of education, age, age squared, sex, and years since the CD4 test.

	BMI		SBP		DBP	
Eligible	−0.956	−1.015	−15.50	−14.12	−11.14	−10.15
	(0.5563)	(0.5296)	(0.0250)	(0.0355)	(0.0195)	(0.0390)
	[−4.150; 2.239]	[−4.191; 2.161]	[−29.03; −1.973]	[−27.27; −0.975]	[−20.47; −1.814]	[−19.78; −0.521]
Eligible *	0.00555	−0.0151	0.297	0.0976	0.181	−0.0318
Deviation	(0.8980)	(0.7335)	(0.2672)	(0.7294)	(0.3249)	(0.8696)
	[−0.0797; 0.0908]	[−0.102; 0.0722]	[−0.230; 0.824]	[−0.459; 0.654]	[−0.181; 0.543]	[−0.414; 0.351]
Obs	241	224	165	155	165	155
Controls	N	Y	N	Y	N	Y
Bandwidth	64	64	40	40	40	40

Eligible* deviation refers to the interaction of the eligibility indicator and the deviation variable. The bandwidths are determined by a data-driven routine introduced in Calonico et al. ^33^. P-values are reported in parentheses, 95% confidence intervals in square brackets.

### Robustness checks

**Figure 4:**
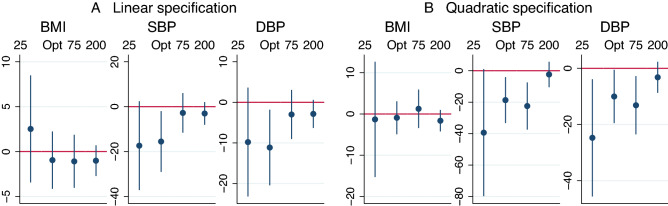
Panel (**A**) depicts the coefficient estimates and 95% CI from Eq. (1) at different bandwidths. The data-driven optimal bandwidth for BMI, SBP and DBP is ± 64, ± 40, and ± 40 CD4 cells/μl, respectively. Panel (**B**) depicts the coefficient estimates and the 95% CIs from estimating Eq. (1) with addition of an quadratic function of the deviation. For comparability, the optimal bandwidth of the linear specification is used. The optimal quadratic bandwidths would be ± 100, ± 80 and ± 85, respectively. Results are robust to the employment of the optimal bandwidth for the quadratic specification.

We assess the robustness of the results by employing different bandwidths and including a quadratic function for the deviation from the threshold. We choose one smaller bandwidth of ± 25, approximately halving the distance to the threshold, one larger bandwidth of ± 75, increasing the distance to the threshold by the same span, and the largest possible symmetric bandwidth of ± 200. The coefficient estimates and 95% confidence intervals are depicted in Fig. 4. As before, there are no significant impacts of early ART eligibility on BMI compared to deferred eligibility. In the linear specification, the coefficient estimates for SBP and DBP in the smaller bandwidth are similar to the estimates in the optimal bandwidth, but not significantly different from zero due to the wider confidence interval. For both, SBP and DBP, the coefficient estimates move towards zero in the two wider bandwidths. In the quadratic specification, the coefficient estimate is significant at a 10% respectively 5% level for SBP and DBP in all bandwidths except for the largest.

### Heterogeneity by sex and year

Next, we test for heterogeneity by sex. As depicted in Table 3, there are significantly negative effects of ART eligibility on SBP and DBP for women, but not for men. However, the effects on the two groups are not statistically different from each other. There are only few observations for men (80% of the individuals within a ± 50 bandwidth are female), such that an effect different from zero or from the effect on women might not be detectable.

**Table 3:** Heterogeneity analysis by sex, using a linear specification. Coefficients are the sum of the base treatment effect and the respective group interaction effect.

	BMI	SBP	DBP
Male	0.113	−9.441	−8.579
	(0.9573)	(0.3604)	(0.1831)
	[−4.027;4.252]	[−29.770;10.887]	[−21.250;4.093]
Female	−1.365	−16.029	−11.540
	(0.3780)	(0.0210)	(0.0201)
	[−4.408; 1.679]	[−29.609; −2.450]	[−21.243; −1.837]
Observations	241	165	165
Bandwidth	64	40	40
p-value	0.3246	0.3507	0.5066

P-values are reported in parentheses, 95% confidence intervals in square brackets.

**Figure 5:**
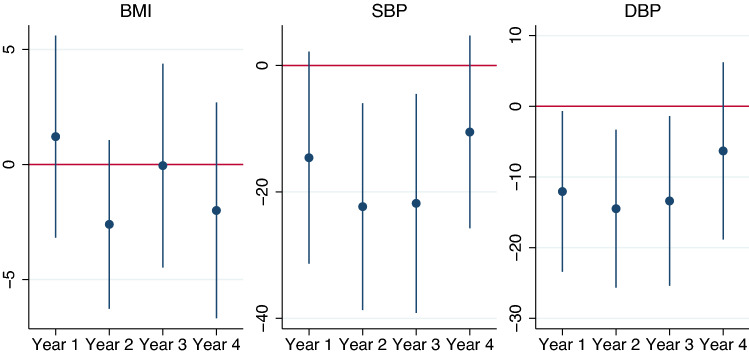
Heterogeneity analysis over time using a linear specification. Coefficient estimates and 95% confidence intervals are depicted. Year 1 depicts the estimates for the eligibility indicator, as year 1 was used as the base level in the regression. Years 2–4 depict the estimates of a linear combination of the base treatment effect plus the interaction effect with the respective year.

Finally, we investigate whether there are heterogeneous effects over time. As depicted in Fig. 5, there is a significant negative effect of early ART eligibility on SBP and DBP in the second and third year, and for DBP also in the first year. The insignificant effect in the fourth year might be caused by several reasons: On the one hand, the treatment effect might diminish or change over time. On the other hand, due to the nature of HIV/AIDS, patients in the initially ineligible group will progress to ART eligibility over time, as their CD4 counts decline, thus attenuating the estimated effect. In total, the yearly effects are statistically not distinguishable from each other.

## Discussion

To our knowledge, our study is the first to identify the causal effects of early ART eligibility on BMI and blood pressure employing a regression discontinuity design. Our approach allows us to compare the BMI and blood pressure of individuals with similar characteristics except for ART eligibility. In contrast to approaches comparing the change in BMI and blood pressure after ART initiation, or comparing BMI and blood pressure across ART naïve and ART individuals, this yields us the causal effect of early compared to deferred ART eligibility at the threshold of 200 CD4 cells/μl.

We do not find any significant effect of early ART eligibility on BMI. However, we find a significant negative effect of early ART eligibility on SBP (− 15.5 mmHg, CI [− 29.03; − 1.973]) and DBP (− 11.1 mmHg, CI [− 27.27; − 0.975]) in a bandwidth of + /− 40 CD4 cells/μl. As a reduction of SBP by 10 mmHg significantly reduces major CVD events^34^, this impact is also clinically relevant. The effects are robust to controls and to using a quadratic function instead of a linear function for the running variable.

The null findings for BMI do not exclude any actual impact of ART on BMI. As BMI might incur substantial measurement error, our sample might not be sufficiently large to detect any changes. Alternatively, as weight gains might emerge over time, our observation period might be too small to identify significant differences to the control group, especially as previously ineligible patients are likely to become eligible sometime after the first CD4 test. Even so, our null results are in line with a meta-analysis for sub-Saharan Africa finding no association between BMI and ART across 36 studies^20^. Nevertheless, previous studies showed that especially stavudine and efavirenz, which were the most common treatment regimes in our sample at that time, can contribute to fat loss^35^. At the same time, initial CD4 counts below 200 cells/μl were significantly associated with weight gains after treatment initiation^36^, which might result in counter-balancing effects on weight. It is possible that this changed over time, as newer ART regimes seem to be associated with more weight gain than older regimes^36^. With the rising prevalence of overweight in southern Africa^37^, any weight gains under newer ART regimes need to be carefully examined to differentiate between a return to a healthy weight and an increase in overweight and obesity^38^.

ART eligibility might decrease blood pressure for several reasons. Firstly, antiretroviral drugs might decrease blood pressure. However, previous evidence suggests that some drug combinations rather increase blood pressure^15,39^. Secondly, the information on ART eligibility and/or the transfer into treatment might alleviate patients’ distress regarding the lethal implications of HIV/AIDS. Quality of Life studies report an increase in the physical but also in the psychological and emotional dimension for patients after ART initiation^40^. The improved well-being could transfer into a stress reduction and hence a decrease of the blood pressure level. Thirdly, the stress-blood pressure link could also work in the other direction: patients informed that they are (just) not eligible for treatment might be faced with a higher fear of dying from AIDS, which could raise their blood pressure. While a systematic literature review indicates a positive correlation between psychological stress and blood pressure, substantial variation in the definition and measurement of stress and blood pressure makes a conclusive assessment of this relationship difficult^41,42^.

Empirically, there is mixed evidence on the link between ART and blood pressure. Globally, a positive correlation is observed after one year of ART, with a progressively stronger correlation over duration of ART^15,17^. There is evidence that an increase in BMI, the type of ART drugs, or both might mediate this relationship^15,39^. For sub-Saharan Africa, no clear association of ART exposure with blood pressure or hypertension is found^19,20^. However, the role of ART duration might matter: an observational study on the same population found a lower blood pressure for ART-exposed PLHIV compared to ART-naïve PLHIV, but a higher blood pressure for PLHIV which have been longer on ART^26^. Thus, our results might be explained by a negative short-run effect of ART eligibility on blood pressure through stress reduction, but a positive long-run effect of ART on blood pressure through either the specific drugs, or weight increases observed in other studies^26,27^.

Our study results should be treated with some caution. First of all, the identification in this regression discontinuity design is purely local, in the sense that it is estimated at the threshold. Hence, the estimated effect cannot be extrapolated to observations further away from the threshold without imposing further assumptions. As guidelines for ART eligibility were shifted to larger CD4 counts and finally lifted, our results explain impacts for patients presenting with comparatively low CD4 counts rather than for patients with comparatively high CD4 counts at the first test. Over time, the CD4 count at first presentation increased in the study region^43^. The introduction of large testing programs over the past decade might have exacerbated this trend further, such that PLHIV are linked to care earlier and present with higher CD4 cell counts nowadays. After 2010, the threshold was increased at first to 350 cells/μl, then to 500 cells/μl, and finally lifted. Investigating the impact at these thresholds would be desirable to assess potential heterogeneous effects at higher CD4 cell count levels, but is not possible with our data, as the anthropometric measures were taken only in 2010. Secondly, the results might not be valid for different study settings. Finally, the share of non-responses in the anthropometric survey evokes the question of representativeness. In our quasi-experimental design, this additionally introduced problems of statistical power, which might be one reason why we cannot identify an impact on BMI.

Investigating the intermediate risk factors BMI, SBP and DBP, we do not find impacts of ART eligibility during the $\le$ 200 cells guideline in KwaZulu-Natal which would contribute to the risk of CVD. In contrast, using a rigorous regression discontinuity design we show that SBP and DBP in early-eligible patients with HIV decreased within the first years compared to patients with HIV with deferred eligibility. The effects of ART might differ under the new guidelines and across a longer time horizon, requiring further assessment of the effects on intermediate risk factors for CVD to evaluate the public health consequences of a growing number of aging PLHIV.

## Data Availability

The data that support the findings of this study are available from the Africa Health Research Institute Data Repository but restrictions apply to the availability of these data, which were used under license for the current study, and so are not publicly available.
